# Active lifestyles in older adults: an integrated predictive model of physical activity and exercise

**DOI:** 10.18632/oncotarget.25352

**Published:** 2018-05-22

**Authors:** Federica Galli, Andrea Chirico, Luca Mallia, Laura Girelli, Michelino De Laurentiis, Fabio Lucidi, Antonio Giordano, Gerardo Botti

**Affiliations:** ^1^ Department of Psychology of Development and Socialization Processes, Sapienza, University of Rome, Rome, Italy; ^2^ Department of Movement, Human and Health Sciences, University of Rome, “Foro Italico”, Rome, Italy; ^3^ Department of Human, Philosophical, Educational Sciences, University of Salerno, Salerno, Italy; ^4^ Breast Department, National Cancer Institute of Naples IRCCS “G. Pascale”, Naples, Italy; ^5^ Sbarro Institute for Cancer Research and Molecular Medicine, Center for Biotechnology College of Science and Technology, Temple University, Philadelphia, PA, U.S.A; ^6^ Department of Medicine, Surgery and Neuroscience, University of Siena, Siena, Italy; ^7^ Division of Pathology, Department of Experimental Oncology, G. Pascale Foundation, National Cancer Institute, IRCCS, Naples, Italy

**Keywords:** older adults, physical activity, exercise, well-being, health, Gerotarget

## Abstract

Physical activity and exercise have been identified as behaviors to preserve physical and mental health in older adults. The aim of the present study was to test the Integrated Behavior Change model in exercise and physical activity behaviors. The study evaluated two different samples of older adults: the first engaged in exercise class, the second doing spontaneous physical activity. The key analyses relied on Variance-Based Structural Modeling, which were performed by means of WARP PLS 6.0 statistical software. The analyses estimated the Integrated Behavior Change model in predicting exercise and physical activity, in a longitudinal design across two months of assessment. The tested models exhibited a good fit with the observed data derived from the model focusing on exercise, as well as with those derived from the model focusing on physical activity. Results showed, also, some effects and relations specific to each behavioral context. Results may form a starting point for future experimental and intervention research.

## INTRODUCTION

In the developed and the developing world, people are living longer. In fact, by the year 2050 the world’s older adult population over age 60 will triple [1]. The major causes of population ageing are the increase of life expectancy and the decline in birth rates in the more developed countries [2]. Italy is one of the oldest countries in the World and the percentage of older adults increased from 20,8% in 2012 to 22,3% in the 2017 and the population’s age mean changed from 43,8 in 2012 to 44,9 in 2017 [3]. The issue of how societies across the globe will view and treat the older adults is gaining attention, with a relevant interest of social and psychological research [4]. Old age is associated with increased risk of several debilitating diseases, such as dementia and cancer, but the most frequent issues faced by individuals aged 65 and over are non - pathological age-related changes, including normal declines in cognition, physical limitations, and loss of partners and friends [5]. So, it is important to facilitate the maintenance of a good quality of life and factors that improve well-being, despite the age-related changes. A recent review showed that active lifestyles reduce the risk of all-cause mortality, prevent various chronic diseases and, in older adults especially, active lifestyles reduce the risk of falls and help to maintain physical function and psychological well-being [6, 7]. In particular, physical activity and exercise have been identified as two important behaviors to target in order to promote physical and mental health [8–10]. First of all, the terms physical activity (PA) and exercise (EX) have been clarified in different studies [11, 12]. PA is defined “as any bodily movement produced by skeletal muscles that results in energy expenditure”[11]. It includes the activity at work, at leisure time or during the household tasks (for example cleaning). Instead, EX is a subcategory of PA that is planned, structured, repetitive, and purposive in the sense that the improvement or maintenance of one or more components of physical fitness [11].

Despite the positive effects of EX and PA are well established, the data of older people who practice these behaviors showed that not many older adults are involved in those. Data collected by WHO [13] showed that globally 1 in 4 adults is not active enough. According to national data in the years 2010-2013, only 24% of older adults (aged 60+ years) met the recommended EX and PA levels.

Different theories have been used in order to evaluate older adults’ active behaviors and to guide interventions that promote EX or PA [14, 15].

Considerable among many of these theories are the Self-Determination Theory (SDT) [16], the Theory of Planned Behavior (TPB) [17] and the Health Action Process Approach (HAPA) [18].

The SDT aims to identify the contextual and environmental factors that can increase or decrease individual motivation. Central to the theory is the distinction between two main types of motivation: intrinsic and extrinsic [19]. Intrinsic motivation pertains to engagement in a specific activity for the pleasure and satisfaction. In contrast, extrinsic motivation refers to activities that are performed to obtain separable outcomes [20]. These motives vary along a continuum: at the lowest end there is the amotivation (when an individual didn’t motivate at all), and the intrinsic motivation is at the highest end [21]. SDT includes different types of regulations determining extrinsic motivation, each with unique characteristics: external (i.e. motivated by rewards or punishments), introjected (i.e. motivated by feeling of guilty) identified (i.e. there are important goals related to the activity) and integrated (i.e. the activity is part of who you are). SDT states that intrinsic motivation can be promoted through autonomy supportive behavior offered by significant figures in the socio-contextual environments in which the individual is engaged, as shown in different literature studies [22, 23]. SDT has been applied especially to health behaviors both in the EX or PA contexts [20, 21, 24]. In particular, some studies demonstrated the relationship between the constructs of self-determination theory and the intention to engage EX and PA, in older adults [25, 26]. In his review, Teixeira *et al.* (2012) demonstrated that motivation differentiates activity levels, specifically, an increase of intrinsic and self-determined motives is positively associated with more PA intention with perceived autonomous fostering the intrinsic motivation.

The TPB [17] is a specific version of the more generalized integrated behavioral model of reasoned action approach [27]. Central to this theory is the idea that the performance of one behavior is determined by behavioral intention. In turn, behavioral intention is determined by three belief-based social cognition behaviors: attitudes (favorable – unfavorable evaluations of the behavior), subjective norms (social pressure to perform the behavior) and perceived behavioral control (the beliefs people hold about resources they have to enact the behavior, and their capacity to overcome behavior related barriers). A large number of reviews and meta-analysis studied the relationships between TPB constructs and PA [28–30]. Results of these studies showed people are more likely to intend to engage PA behavior if they are positively disposed toward it (attitudes), if they perceive social pressure to do so (subjective norms), and if they believe they will be successful (perceived behavioral control). In this sense, Lucidi *et al.*, 2006 [31] examined the TPB in relation to PA behaviors in older adults and showed that the three TPB constructs are significantly correlated with behavioral intention, and these results were confirmed by a recent literature review [32]. A prominent critique of TPB is the imperfect link between intention and behavior engagement. This shortfall in the relationship between intention and behavior has been labeled as “intention-behavior gap”.

A model that explicitly includes post- intentional mediators to overcome the intention-behavior gap is the HAPA. It was originally developed in the late 1980s [33] by the social-cognitive theory [34], the theory of reasoned action [27], and the volition theories of Heckhausen, Gollwitzer, and Kuhl [35] applying this synthesis to the field of health behavior change. The HAPA considers a dual-phase approach: a motivational phase (how individuals form intentions whatever to adopt a behavior) and a volitional phase (how intentions are translated into actual behavior and behavioral maintenance through planning). The HAPA has been applied to numerous healthy behaviors, for example weight loss through diet [22], EX [36] and PA [37]. On this theoretical stream of literature, Maher and Conroy (2016) demonstrated that older adults’ sedentary behavior is negatively associated with planning, and planning has a moderate and positive relationship with intention [38].

Different studies tested whether the integration of these theories would predict health-related behaviors [22, 23, 39, 40]. Recently, Hagger and Chatzisarantis [41] synthesized their theoretical and empirical works on the development of integrated theories of health behaviors. The authors drew these social psychological theories to derive an Integrated Behavior Change (IBC) model, that incorporates the very latest thinking on the psychological influences on behavior change applied to PA behaviors, providing a complementary explanation of the unexplained processes within each theory. The TPB forms the starting point of the proposed IBC model, such that intention represents the most proximal predictor of behavior and mediates the effects of attitudes, subjective norms, and perceived behavioral control on behavior.

A noted limitation of TPB, underlined by the authors, is that the theory is relatively silent on the origins and drivers of the belief-based antecedents of intention [16, 30, 39]. The IBC model proposes the integration of the SDT and TPB, specifically, the model poses the TPB constructs as mediators of the relationship between autonomous motivation and intention. Therefore, in order to overcome the intention-behavior gap, the IBC model introduces the planning construct of HAPA as moderator of the intention-behavior relationship.

The main aim of the present study was to test the IBC model both in EX and PA behaviors on two different samples of older adults, adding perceived autonomy support as predictor of the autonomous motivation.

**Figure 1:**
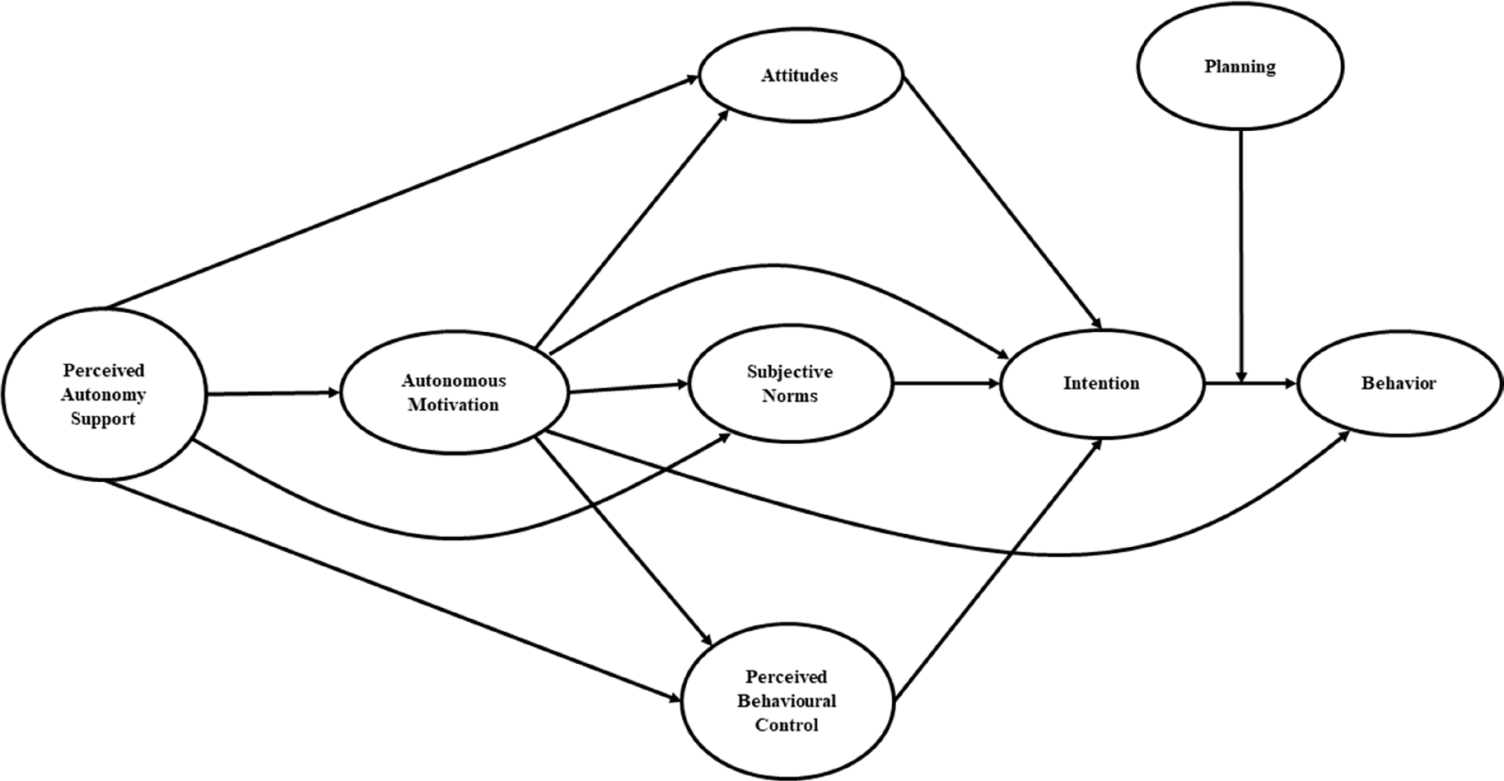
The Integrated Behavior Change model linking perceived autonomy support, autonomous motivation, attitudes, subjective norms, perceived behavioral control, intention and planning on behavior

We expect that the hypothesized model (Figure 1) would fit adequately with both behaviors. According to SDT [16], we hypothesized that higher perceived autonomy support would predict autonomous motivation. Secondarily, according to the IBC model, we hypothesized that motivation in turns will affect both attitudes, subjective norms and perceived behavioral control; the TPB constructs would be related to the intention in enact the behaviors, and higher intention would be related with a higher probability to enact it. Finally, it is also expected that relationship between intention and behavior will be moderated by planning construct of HAPA.

## RESULTS

### Socio demographic differences on PA and EX behavior

The EX sample comprised 192 older adults who completed Time 1 and Time 2 questionnaires (69.8% female; mean _age_= 71.13, years SD = 6.58, range 60 – 88), the PA sample comprised 100 older adults who completed Time 1 and Time 2 questionnaires (62.0 % female; mean _age_= 75.78 years, SD = 7.53, range 62 – 94). Univariate analyses of variance on age, gender distribution, and all the key variables measured showed no significant differences between participants filled out both Time 1 and Time 2 assessments and those that dropped out after Time 1 in both samples. Zero-order correlations between age and behavior were not statistically significant in both contexts. Univariate variance analysis evaluating the effect of gender on behavior showed a statistically significant difference only in PA samples, with males slightly more likely to do EX (PA sample F _(1,99)_ = 5.64; *p* = .02 partial eta^2^ = .05).

The descriptive statistics of the study variables for both the samples are reported in Table 1 along with the zero-order correlations and the Cronbach’s alpha of the measures used.

**Table 1:** Descriptive statistics, reliability and inter-correlation among the key variables of the study

	Mean (SD)	Cronbach’s Altpha	Correlations								
			1	2	3	4	5	6	7	8	9
*1) Perceived Autonomy support*											
EX	6.54 (.58)	.92	-								
PA	5.86 (1.31)	.93	-								
*2) Autonomous motivation (RAI)*											
EX	16.06 (5.57)	.78	.13	−							
PA	11.63 (7.07)	.79	.46^***^	−							
*3) Attitudes*											
EX	6.70 (.75)	.88	.18^*^	.26^***^	−						
PA	6.29 (.91)	.93	.43^***^	.51^***^	−						
*4) Subjective Norms*											
EX	5.95 (1.38)	.91	.04	.07	.28^***^	−					
PA	5.89 (1.49)	.91	.47^***^	.42^***^	.55^***^	−					
*5) Perceived Behavioral Control*											
EX	6.44 (.97)	.80	.20^**^	.39^***^	.56^***^	.22^**^	*−*				
PA	5.66 (1.31)	.67	.38^***^	.56^***^	.43^***^	.47^**^	−				
*6) Intention*											
EX	6.51 (1.06)	.99	.18^*^	.33^***^	.45^***^	.25^**^	.67^***^	−			
PA	5.64 (1.72)	.96	.45^***^	.58^***^	.67^***^	.61^**^	.61^***^	−			
*7) Planning*											
EX	5.58 (1.50)	.95	.14^*^	.29^***^	.40^***^	.34^***^	.61^***^	.64^***^	−		
PA	4.09 (1.73)	.94	.11	.22^*^	.37^***^	.27^**^	.30^**^	.50^***^	−		
*8) Behavior (Time 1)*											
EX	2.56 (.87)	-	-.09	.21^**^	−.04	−.22^**^	.13	.17^*^	.16^*^	−	
PA	22.08 (19.43)	-	.22^*^	.40^*^	.38^***^	.30^**^	.43^***^	.45^***^	.20	−	
*9) Behavior (Time 2)*											
EX	2.26 (1.05)	-	-.04	.22^**^	.14	−.05	.30^***^	.30^***^	.26^**^	.56^***^	−
PA	25.35 (20.29)	-	.31^**^	.40^*^	.37^**^	.22^*^	.38^**^	.38^***^	.21^*^	.70^**^	−

### Preliminary analyses for validity criteria

Measurement-level statistics of the VB-SEM of the model data were examined to ensure whether the latent variables met construct and discriminant validity criteria. Reliability coefficients exceeded the .700 criterion for the factors included in both the models. In all cases, the square root of the AVE for each latent variable exceeded the correlation between all the variables. Composite reliability coefficients, AVE for the factors, and factor intercorrelations are available upon request.

### Fit of the models on EX and PA samples

Overall, as reported in Table 2, the hypothesized models exhibited a good fit with the observed data derived from the model focusing on EX, as well as with those derived from the model focusing on PA. The good fit of the hypothesized models was confirmed also controlling for the past behavior, both in the EX and in the PA model.

**Table 2:** Goodness of fit indexes of structural equation modelling of the hypothesized model for each sample

	Exercise Sample	Physical Activity Sample
GoF	.412 [.436]	.554 [.564]
APC	.218; *p* < .001 [.207; *p* < .001]	.291; *p* < .001 [.252; *p* <. 001]
ARS	.195; *p* = .001 [.215; *p* < .001]	.361; *p* < .001 [.367; *p* < .001]
AFVIF	1.242 [1.981]	1.468 [1.961]

Note. VB-SEM goodness-of-fit indexes controlling for past behavior measured at Time 1 are in square parentheses.

### The application of the IBC model in PA and EX behavior

**Figure 2:**
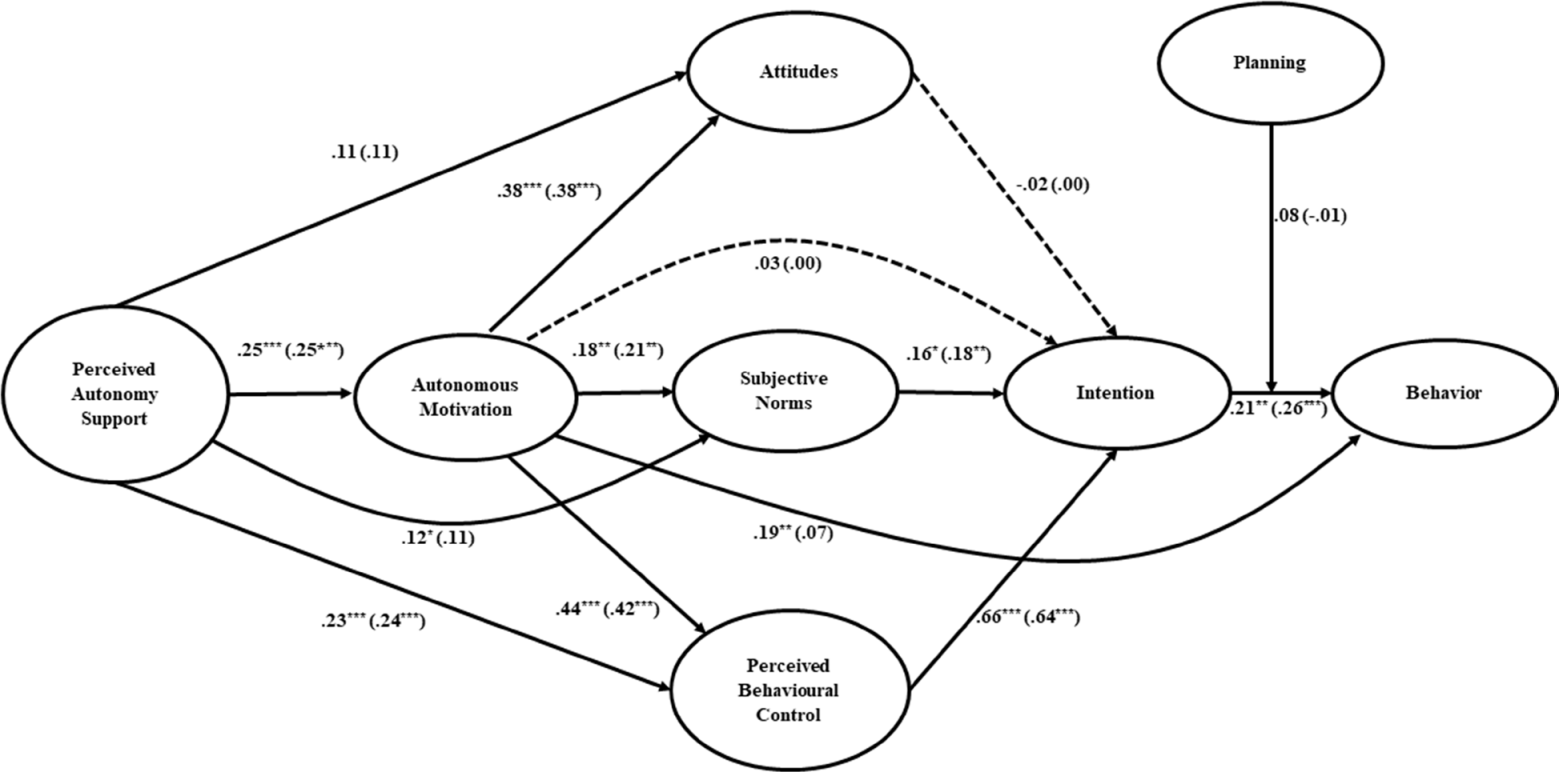
The Integrated Behavior Change model linking perceived autonomy support, autonomous motivation, attitudes, subjective norms, perceived behavioral control, intention and planning on Exercise behavior Note Standardized path coefficients for the structural equation model estimated controlling for behavior measured at Time 1 are reported in parentheses. The effects of past behavior measured at Time 1 on each variable in the model figure were omitted for clarity. These paths were freely estimated in the VB-SEM analysis but not depicted in diagram: past behavior → perceived autonomy support (β = –.15, *p* = .017); past behavior → autonomous motivation (β = .21, *p* = .001); past behavior → attitude (β = –.09, *p* = .11); past behavior → subjective norm (β = –.32, *p* < .001); past behavior → perceived behavioral control (β = .06, *p* = .207); past behavior → intention (β = .13, *p* =.03); past behavior → planning (β = .22, *p* < .001); past behavior → behavior at Time 2 (β = .49, *p* < .001). Dashed lines indicate paths that were not statistically significant (*p* > .05) in the SEM analysis without controlling for past behavior. ^***^p < .001; ^**^p < .01; ^*^p < .05.

Focusing on the test of the hypothesized effects in the model on the EX (Figure 2), perceived autonomy support was statistically significant predictor of the autonomous motivation. Furthermore, perceived autonomy support predicted directly and significantly only subjective norms and perceived behavioral control variables. There was a significant positive effect of autonomous motivation on all the TPB constructs (i.e., attitudes, subjective norms and perceived behavioral control), of those measures only subjective norms and perceived behavioral control were significantly related to the intention. Autonomous motivation showed also a direct effect on behavior. Finally, intention significantly predicted the behavior, but this relationship was not significantly moderated by the planning.

Results of indirect effects of the model (Table 3) showed an indirect effect of the perceived autonomy support through the autonomous motivation upon attitudes and perceived behavioral control. The autonomous motivation, in turn, showed a significant indirect effect on intention only through the perceived behavioral control.

**Table 3:** Standardized path coefficients for mediated effects for the structural equation models for each behaviour

Paths	Behaviour	Mediator	Direct Effect	Indirect effect	Total effect	Mediation
Perceived Autonomy Support → Attitudes	EX	Relative Autonomy Index	.11	.10^*^	.21^**^	Yes
	PA		.32^***^	.20^**^	.52^***^	Yes
Perceived Autonomy Support → Subjective Norms	EX	Relative Autonomy Index	.12^*^	.05	.17^*^	No
	PA		.33^***^	.15^*^	.48^***^	Yes
Perceived Autonomy Support → Perceived Behavioural control	EX	Relative Autonomy Index	.23^**^	.11^*^	.34^***^	Yes
	PA		.14	.29^***^	.43^***^	Yes
Relative Autonomy Index → Intention	EX	Attitude	.03	-.01	.02	No
	PA		.07	.19^**^	.26^**^	Yes
Relative Autonomy Index → Intention	EX	Subjective Norms	.03	.03	.06	No
	PA		.07	.05	.12	No
Relative Autonomy Index → Intention	EX	Perceived Behavioral Control	.03	.29^***^	.31^***^	Yes
	PA		.07	.12^*^	.19^*^	Yes

^***^p < .001; ^**^p < .01; ^*^p < .05.

As resulted in Figure 2 (i.e. the values in brackets), controlling for past behavior, the model on the EX resulted almost in an identical path estimation as the previous one. The paths linking perceived autonomy support to subjective norms, and autonomous motivation to behavior, turn to be non-significant. The inclusion of past behavior on the model further resulted in an improvement of the behavior’s variance explained by the model (from R^2^ = .10 to R^2^ = .40).

**Figure 3:**
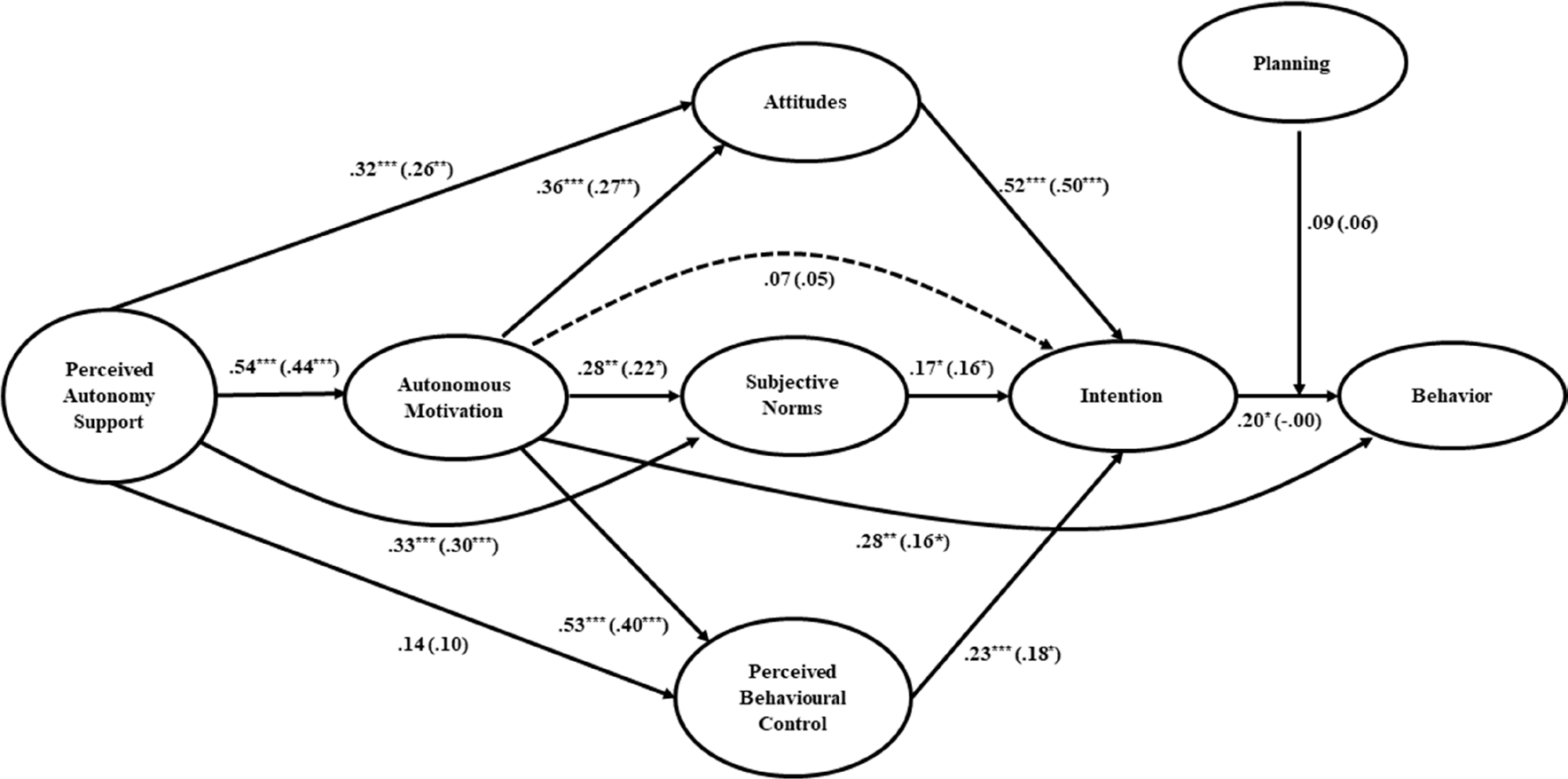
The Integrated Behavior Change model linking perceived autonomy support, autonomous motivation, attitudes, subjective norms, perceived behavioral control, intention and planning on Physical Activity behavior *Note*: Standardized path coefficients for the structural equation model estimated controlling for behavior measured at Time 1 are reported in parentheses. The effects of past behavior measured at Time 1 on each variable in the model figure were omitted for clarity. These paths were freely estimated in the VB-SEM analysis but not depicted in diagram: past behavior → perceived autonomy support (β = .31, *p* < .001); past behavior → autonomous motivation (β = .31, *p* < .001); past behavior → attitude (β = .28, *p* < .001); past behavior → subjective norm (β = .18, *p* = .032); past behavior → perceived behavioral control (β = .31, *p* < .001); past behavior → intention (β = .14, *p* =.07); past behavior → planning (β = .25, *p* =.005); past behavior → behavior at Time 2 (β = .65, *p* < .001). Dashed lines indicate paths that were not statistically significant (*p* > .05) in the SEM analysis without controlling for past behavior. ^***^p < .001; ^**^p <.001; ^*^p <.05.

Testing the model dealing with PA behavior, (Figure 3) results showed very similar results to the EX model. In fact, perceived autonomy support was statistically significant related to autonomous motivation as well as to attitudes and subjective norms. There was a significant positive effect of autonomous motivation on TPB constructs (attitudes, subjective norms and perceived behavioral control), that in turn were significantly related with the intention. Also in this sample, autonomous motivation showed a direct effect on behavior. Finally, intention significantly predicted the behavior and this path was not moderated by the planning.

Furthermore, considering the indirect effects, as reported in Table 3, the perceived autonomy support revealed significant indirect effects through the autonomous motivation upon all the three constructs of TPB. In turn, the autonomous motivation indirectly affects the intention only through attitudes and perceived behavioral control.

As showed in Figure 3 (i.e. the values in brackets), even for PA model, controlling for past behavior the model resulted almost in an identical path estimation with respect to the previous one. However, to note that the effect of the intention on behavior became substantially not statistically significant. Overall, even in this case, the inclusion in the model of past behavior resulted in an improvement of the behavior’s variance explained by the model (from R^2^ = .17 to R^2^ = .53).

## DISCUSSION

The purpose of the present study was to test the IBC model [41], evaluating also the perceived autonomy support as predictor of motivation, in two healthy behaviors: EX and PA, on two samples of older adults.

Findings from the fit indexes confirmed a good fit of data with the hypothesized IBC model in both the behavioral domains. In both the samples there were several consistent patterns of effects in accordance with the expected patterns of the IBC model. Specifically, as hypothesized, results showed perceived autonomy support significantly increases autonomous motivation, that in turn resulted a significant predictor of attitudes, subjective norms and perceived behavioral control, then subjective norms and perceived behavioral control were significant predictors of intention. Moreover, intention was a significant predictor of behavior in both behavioral contexts. There were, also, some important mediation effects in the two contexts. In fact, the effect of perceived autonomy support on attitudes and on perceived behavioral control was mediated by autonomous motivation. Furthermore, the relationship between motivation and intention was mediated by the perceived behavioral control.

Overall, from our data it seems that, in line with the SDT, when individuals were provided with forms of support of their need to feel autonomous, they are more likely to be intrinsically motivated to enact that behavior than individual with poor support. Our results, then, suggest that individuals’ perception of significant others creating a supporting environment for their autonomy not only fosters their autonomous motivation, but it is also positively associated with their beliefs (attitudes, subjective norms and perceived behavioral control) toward that behavior. This is in line with the IBC model [41] and very similar to other researches’ results that have shown significant relations between the immediate antecedents of intention for the TPB theory and the autonomous form of motivation from SDT [22, 41].

According with the IBC model, the moderation role of the planning in the intention-behavior relationship has been evaluated. Literature on this topic showed controversial results providing evidence for a moderation role of the planning construct [42], as well as for a mediation [22], or a moderated mediation [43] of the intention-behavior relationship. Our results showed that this moderation effect was not present at a model level, even if zero-order correlations showed bivariate relationship between planning with both intention and behavior. In any case, these results are consistent with previous studies showing that planning is not a significant moderator when trying to maintain a behavior that is already performed, as it reflects EX and PA in our sample of older adults [22, 44].

It is important to note that there were some effects and relations specific to each behavioral context. The direct effect of perceived autonomy support on perceived behavioral control was significant in the EX domain but not in the PA one, conversely its effect on attitudes was present only considering PA context and not considering the EX behavior. Moreover, the effect of attitudes on intention was significant only in the PA context. Also, some mediation effects were specific for only one of the two contexts, in example, the relation between perceived autonomy support and subjective norms was mediated by autonomous motivation only in the PA context, then the relation between motivation and intention was mediated by attitudes only for the PA behavior as well. The contexts’ specificity of some of these patterns seem to reply patterns found also in other studies that used similar models [22, 39, 40, 45].

Finally, we controlled for past behavior measured at Time 1 by including it as predictor of all variables in the model. We found that the patterns of relationships were consistent with those estimated without controlling for past behavior, although we found some differences related to the specific behavioral context. In fact, for the EX context controlling for past behavior showed a substantial similar model in terms of effects, with very slight differences, with past behavior related to future behavior with a moderate effect and a general improvement of the variance explained by the model. In the PA context, instead, we found a substantial attenuation of the effects for most of the model paths, in particular, a complete attenuation, resulting in a non-significant effect, for the intention-behavior relationship, with the past behavior strongly and significantly related to the future behavior as well as with a substantial improvement of the variance explained by the model. These results are in line with previous researches [46–49].

The variability in the effects of past behavior in the two-behavioral domain is in line with previous research [22]. From a theoretical point of view, it demonstrates the efficacy of the IBC model in accounting for variance in future behavior once the effects of past behavior have been controlled. PA resulted in a stable personal disposition that has strongly consistency over the time, and led the past behavior acting as an automated process. Conversely, participating in an exercise course needs to be fostered through a more intrinsic, complex, cognitive process relating several variables explained by the IBC model.

From a practical perspective, there is an opportunity for many professionals who deal with older people in order to promote any PA behaviors. The study, in fact, provides insightful results suggesting general or specific applicable approaches for the two behavioral contexts. In this sense, supporting the autonomous perceptions of older adults would increase their intrinsic motivation in both the contexts, and this would be a boost for realizing their intention in enact PA or EX behavior through their beliefs system. This information should be considered in designing program aimed to activate or maintain the intentions toward PA or EX through the key variables of the IBC model specifically related to each behavior.

There are two possible limitations that should be addressed in future research: the data are limited to a targeted sample and could be not generalizable to the population of older adults. The design of the study did not permit us to identify causal relationship between the key variables of the study. Despite these limitations, present results support the important relations within the key variables of IBC model integrating the TPB the SDT and the HAPA. Future researches are needed in order to test the model in different target populations accounting also for reciprocal relations among constructs, also clarifying the role of planning in the intention-behavior gap.

This research is the first attempt in applying the IBC model in its original theorization to the PA in two samples of older adults. Results may form a starting point for future experimental and intervention research. Secondarily, this is one of few studies including specific behaviors related to physical activity (EX and PA) in a similar target group (older adults).

## METHODS

### Study population

The study relied on two samples of older adults. Participants were selected in order to consider the two different behaviors (EX and PA).

The EX sample comprised 222 older adults (69.8% females; mean age: 71.14 years; SD = 6.47; range: 60–88) recruited in three fitness centers in Rome, Italy. Inclusion criteria for this sample (EX sample) were: being older than 60 and declaring attending regularly in an exercise class group, being in a good clinical condition. Attrition rate across the two times of data collection due to absences or inability to match the data was 13.5% (*N* = 30) leaving a total of 192 subjects.

The PA sample comprised 133 older adults (62.2% females; mean age: 75.29 years; SD = 7.41; range 62–94) recruited in two senior centers in Rome, Italy. Inclusion criteria for this sample (PA sample) were: being older than 60, declaring a regular PA as unique form of activity and being in a good clinical condition. Thirty-one older adults (23.3%) were excluded because declared EX as additional form of activity over the PA, leaving a total of 102 older adults meeting all the inclusion criteria. Attrition rate across the two times of data collection due to absences or inability to match the data was 1.5% (*N* = 2) leaving a total of 100 subjects. Each target behavior was defined for the participants in a standardized set of instructions. The present study is configured as a prospective study with two data collection time. In the first assessment time, participants of both samples filled a booklet of self-reported questionnaires measuring the key factors of the IBC model, as well as self-reported past behavior.

During the second assessment, conducted two months later, we asked the participants of both sample to self-report their behavior in the last two months.

All recruited persons gave their consent to participate in the study. The study was approved by the Ethics Review Board of [UNIVERSITY OMITTED FOR MASKED REVIEW]. Participants were duly informed about the aims and purposes of the study and about their participation rights (e.g., confidentiality of responses, right to withdraw any time without any consequences).

### Measures

Behavior specific version of each measure was adapted for the target behavior according to the case or specifically developed from the component theories of the adopted integrated model based on previous researches [17, 22, 50]. All scales were translated from English to Italian. The translation was conducted by two English-Italian bilinguals using standardized back translation procedures [51].

Perceived Autonomy Support was measured by Perceived Autonomy Support Scale for Exercise Setting [52]. PASSES measures autonomy support from an important significant source that is likely to perform one’s exercise behavior. The scale has been validated with three different salient sources (e.g. physical education instructor, peers, parents) showing in both consistent and discriminant validity. The measure comprises 12 items with 7-point Likert-type response scales with the “strongly disagree” (1) and “strongly agree” (7) endpoints. Higher scores per PASSES scales reflect greater perceptions of autonomy support. In particular, in the EX sample of this study, we considered participants’ trainer as the source of autonomy support (e.g. “My trainer listens to me about my exercise habits”).

In the PA sample, according to other studies [53], the scale was adapted requesting the participants to nominate one important other who had the greatest impact on their PA behaviors, who was considered as source of autonomy support for the scale. (e.g. “My important other listens to me about doing PA”).

Autonomous Motivation was measured in the EX sample of the study using the Behavioral Regulation in Exercise Questionnaire, (BREQ-3) version 3 [54]. The scale was presented with the following stem: “Why do I exercise?”. Participants were asked to answer on a 5-point Likert scale from “not true for me” (0) to “very true for me” (4) endpoints. The BREQ-3 comprises 24 items and six factors (each of 4 items): amotivation (e.g.: “I don’t see the point in exercising”); external regulation (e.g.: “I exercise because other people say I should”); introjected regulation (e.g.: “I feel guilty when I don’t exercise”); identified regulation (e.g.: “I value the benefits of exercise”); integrated regulation (e.g.: “I consider exercise a fundamental part of who I am”); intrinsic motivation (e.g.: “I exercise because it’s fun”). In order to maximize the parsimony of the model of our study, the relative autonomy index (RAI) [50] was calculated. RAI is a single score derived from the subscales that gives an index of the degree to which respondents feel self-determined. Items from the intrinsic motivation subscale were assigned a weight of +3, integrated regulation items a weight of +2, identified regulation items a weight of +1, introjected regulation items a weight of −1, external regulation items a weight of −2, and amotivation items a weight of −3. Each subscale score is multiplied by its weighting and then these weighted scores are summed. Higher, positive scores indicate greater relative autonomy; lower, negative scores indicate more controlled regulation.

Participants of the PA sample completed a modified version of the BREQ-3 [55, 56]. The modification consisted of the replacement of the word “exercise” with the word “physical activity”.

Attitudes were measured in the EX sample using a scale developed by the authors, following the recommendations of Ajzen (1991) for TPB constructs development, and based on measures used in previous studies [22, 44]. The scale, introduced by “I think exercising for the next two months, would be…”, comprised six items with responses provided on seven-points semantic differential scales with the bipolar adjectives: ‘bad–good’, ‘harmful–beneficial’, ‘unenjoyable–enjoyable’, ‘useful–useless’, ‘foolish–wise’ and ‘unpleasant–pleasant’.

Participants of PA sample completed a similar version of the scale introduced by “I think doing physical activity for the next two months, would be…”.

Subjective Norms were measured in the EX sample using a scale developed by the authors, following the recommendations of Ajzen (1991) for TPB constructs development and based on measures used in previous studies [22, 44]. Three items measured participants’ subjective norms by asking respondents to indicate on a 7-point Likert scale from “strongly disagree” (1) and “strongly agree” (7) endpoints to what extent meaningful others: “would like me to do exercise for the next two months”; “would consider good for me to do exercise for the next two months”; “would appreciate me to do exercise for the next two months”. Item scores were aggregated into a single score, for which higher values indicated greater normative social pressure toward the behavior.

Participants of PA sample completed a similar version of the scale consisting in the replacement of the word “exercise” with the word “physical activity” in all instances.

Perceived Behavioral Control was measured in the EX sample using a scale developed by the authors, following the recommendations of Ajzen (1991) for TPB constructs development and based on measures used in previous studies [22, 44]. Three items measured participants’ perceived behavioral control by asking respondents to indicate on a 7-point Likert scale (e.g. “I’m confident I can exercise over the next two months”). Item scores were aggregated into a single score, for which higher values indicated greater perceived confidence toward the behavior.

Participants of PA sample completed a similar version of the scale consisting in the replacement of the word “exercise” with the word “physical activity” in all instances.

Intention was measured in the EX sample using a scale developed by the authors, following the recommendations of Ajzen (1991) for TPB constructs development and based on measures used in previous studies [22, 44]. Four items measured participants’ intention by asking respondents to indicate on a 7-point Likert scale from “strongly disagree” (1) and “strongly agree” (7) endpoints, (e.g. “I intend to exercise over the next two months”). Item scores were aggregated into a single score, for which higher values indicated greater intention toward the behavior.

Participants of PA sample completed a similar version of the scale consisting in the replacement of the word “exercise” with the word “physical activity” in all instances.

Planning was assessed in the exercise sample using the action and coping planning independent measure validated by Sniehotta *et al.*, (2005) [57]. Four items measured action planning (e.g., “I’ve already planned how I will exercise”) and four measured coping planning (e.g., “I’m going to make a detailed plan about how to cope with possible setbacks that can impede me to exercise”). Participants were asked to response on 7-point Likert scale ranging from “not true at all” (1) to “very true” (7) endpoints.

Participants of PA sample completed a similar version of the scale consisting in the replacement of the word “exercise” with the word “physical activity” in all instances.

Self-reported behavior was measured at the first wave and at the second wave of data collection, two months later.

In the EX sample, it was requested the weekly attendance to the center.

In the PA sample, self-reported behavior was measured by Godin-Leisure Questionnaire [58]. The Godin-Leisure Questionnaire assesses the frequency of physical activity completed during free time for at least 15 minutes over a typical week. This measure includes three open-ended items that measure the frequency of strenuous (e.g., jogging), moderate (e.g., fast walking), and light (e.g., easy walking) activity. The weekly activity was weighted for the type of the activity: 3 per light activity, 5 per moderate activity and 9 per strenuous one. The sum of weighted light, moderate and strenuous physical activity gave the total self-reported behavior in minutes per week.

### Data analysis

The key analyses of the present study relied on Variance-Based Structural Modeling (VB-SEM – also known as Partial Least Squares analysis), which were performed by means of the WARP PLS v.6.0 statistical software [59]. These analyses overall tested and estimated the hypothesized IBC model (Figure 1) linking perceived autonomy support, autonomous motivation, attitudes, subjective norms, perceived behavioral control, intention and planning in predicting two different types of behaviors (i.e., EX and PA) across two months.

VB-SEM is similar to a covariance-based SEM analyses, it explicitly models measurement error through the construction of latent factors. However, unlike methods used in covariance-based SEM, the partial least-squares algorithm is based on ranked data and is, therefore, distribution-free (i.e., the estimation is less affected by the complexity of the model, small sample size, or non-normality of the data). VB-SEM analysis permits the evaluation of the model at the measurement level and at the structural level according to published criteria for VB-SEM models [60]. At the measurement level, VB-SEM establishes construct validity of the latent factors using the average variance extracted (AVE) and the composite reliability coefficients (ρ), which should exceed .50 and .70, respectively. Discriminant validity is supported when the square-root of the AVE for each latent variable exceeds its correlation coefficient with other latent variables [60]. At the structural level, VB-SEM establishes adequacy of the hypothesized pattern of relations among the model constructs using an overall goodness-of-fit (GoF) index given by the square root of the product of the AVE and average R^2^ for the model (.100, .250, and .360 correspond to small, medium, and large effect sizes) [61]. This method, therefore, provides further information on the adequacy of the model by the average path coefficient (APC) and average R^2^ (ARS) coefficient across the model, both of which should be statistically significant different from zero. Furthermore, it checks the potential for multicollinearity using the full collinearity variance inflation factor (AFVIF) with values lower than 3.300 indicative of no issues with multicollinearity [59].

Finally, in order to control for past behavior effect on all the variables, a further analysis of the data that included behavior measured at Time 1 as a control variable has been conducted [45].

## Abbreviations

PA: Physical Activity
EX: Exercise
SDT: Self Determination Theory
TPB: Theory of Planned Behaviour
HAPA: Health Action Process Approach
IBC: Integrated Behavior Change
PLS: The Partial Least Squares
VB-SEM: Variance-Based Structural Modeling
AVE: Average Variance Extracted
SD: Standard Deviation
PASSES: Perceived Autonomy Support Scale for Exercise Setting
BREQ: Behavioral Regulation in Exercise Questionnaire
RAI: Relative Autonomy Index
GoF: Goodness-of-Fit
APC: Average Path Coefficient
ARS: Average R-squared
AFVIF: Average Variance Inflation Factor
